# Supplementary figures and images for: Exploring association between place of delivery and newborn care with early-neonatal mortality in Bangladesh
Source: PLoS One. 2022 Jan 27;17(1):e0262408. doi: 10.1371/journal.pone.0262408 (PMC8794140; doi:10.1371/journal.pone.0262408)

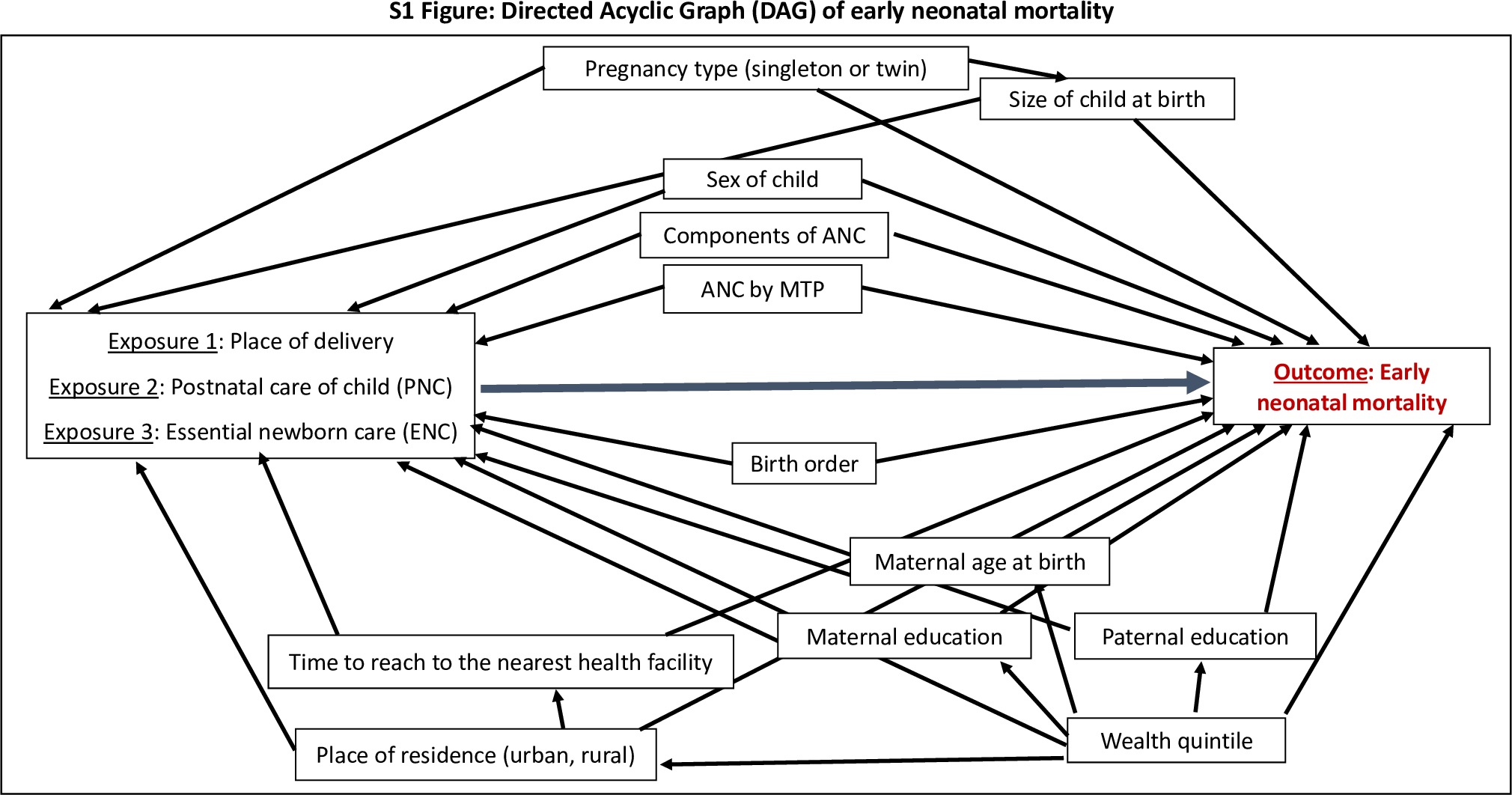

Supplement: S1 Fig — (TIF) [file pone.0262408.s001.tif]
