# Supplementary material for: Exploring association between place of delivery and newborn care with early-neonatal mortality in Bangladesh
Source: PLoS One. 2022 Jan 27;17(1):e0262408. doi: 10.1371/journal.pone.0262408 (PMC8794140; doi:10.1371/journal.pone.0262408)
Supplement: Attachment — Submitted filename: ReviewReport_PONE-D-20-30510.pdf [file pone.0262408.s003.pdf]

Review Report on **The role of institutional delivery and newborn care in preventing early-neonatal mortality in Bangladesh**

The manuscript deals with identifying the factors affecting early neonatal mortality (ENNM), which represents more than 80% of all neonatal mortality in Bangladesh, with a specific focus on place of delivery and newborn care. The 2014 Bangladesh Demographic and Health Survey data were used for assessing early-neonatal survival in children born in the three years preceding the survey. Multivariate logistic regression models have been used. The study findings highlight the importance of newborn and postnatal care in preventing early neonatal deaths.

I suggest some revisions. Following questions must be needed to answer.

1. Any variables about husband have not been used. Particularly, husband's education, or parrental education, employment status, occopation are important to consider. Besides, mother's age at first birth is important.
2. Why 2018 BDHS data has not been used for this study instead of BDHS 2014 data?
3. How about the death rate among the missing cases? Total 309 cases have been omitted. Just in case if considerable amount of death rate is found among 309 cases, then suitable imputation method could be used to impute the missing observations.
4. Have the twin babies considered here. If so then, whether it could be an important factor in the model.
5. In Statistical Analysis section, Variance inflation factors (VIF) were assessed to examine collinearity between variables before entering them into the multivariable models? What cut off point was used here?
6. The confounding could be existed here. Particularly, ANC visits and ENNM might be under important consideration in making such unobserved confounding. What is the response of authoors in this regards?
7. Is simple map as displayed in Figure 1 important? Rather authors could have mapping for Bangladesh by disaggegating to division levels. Authors could use many software including "mapReasy" R package (Islam et al., 2017).
