# Supplementary material for: Exploring association between place of delivery and newborn care with early-neonatal mortality in Bangladesh
Source: PLoS One. 2022 Jan 27;17(1):e0262408. doi: 10.1371/journal.pone.0262408 (PMC8794140; doi:10.1371/journal.pone.0262408)
Supplement: Attachment — Submitted filename: ReviewReport_PONE-D-20-30510R1.pdf [file pone.0262408.s005.pdf]

Review Report on **The role of institutional delivery and newborn care in preventing early-neonatal mortality in Bangladesh**

I have gone through the revised manuscript and found insufficient responses to some vital points. Hence, still following questions must be responded properly to improve this manuscript.

1. Authors need to give more strong reference against their claim “Based on best practice, our preference is to include only the minimally sufficient adjustment set" as mentioned in the answer of first point. Many studies found direct associations of parental education, employment status, occupation with neo-natal mortality. Otherwise, they need to carry out the analysis with these variables in the model.
2. The data of BDHS2018 is available and all the variable codes are same as BDHS2014. Authors must need to redo all analyses with this data. Authors mentioned that they will do the same analysis and make another paper which is pointless as the dataset is already available.
3. Authors did not mention whether there was no any missingness in the covariates considered apart from answering missingness among the discarded 309 cases.
4. Figure 1 in previous manuscript was suggested to revise by using MapReasy which is important to strengthen this manuscript but authors ommitted this. Authors need to generate and innclude such graph.
5. Authors still mentioned in abstract and statisticcal analysis section that multivaiate logistic..... It should be multivaiable logistic....
6. Some references are missing pages, volume, series etc. E.g., references 18, 36, 41, 43.
7. Study strengths were unnecessarily been mentioned in the last section. Only limitations are OK.
8. Page 6, 112: DAG has been created and a minimally sufficient adjustment is set according to ref 27. There are many published well known studies but authors cited ref 27 which is not a published paper and not worthy. Here how had DAG been created?
9. Data source is insufficiently discussed.
