# Supplementary material for: Exploring association between place of delivery and newborn care with early-neonatal mortality in Bangladesh
Source: PLoS One. 2022 Jan 27;17(1):e0262408. doi: 10.1371/journal.pone.0262408 (PMC8794140; doi:10.1371/journal.pone.0262408)
Supplement: Attachment — Submitted filename: Review.pdf [file pone.0262408.s006.pdf]

The authors have made some significant changes. However, there are still many major issues that need to be addressed:

1. In response to aggregating 2/3 cycles of data to improve statistical power, the authors mentioned that the proportion of women delivering in health facilities has been increasing over time, affecting the relationship between delivery care and early neonatal mortality. If this is true, why would we believe findings from BDHS 2014 data would reflect the current scenario of the association? The BDHS 2017-18 dataset is already publicly available, and it should only take a couple of days to access the dataset. Then why would be we believe BDHS 2014 dataset is more likely to reflect the current scenario than BDHS 2017-18?

   In Table 4 (model 3), the authors adjusted the model for many covariates. However, the number of events is only 69 in the study (Table 1). For example, there are only 69/29 ≈ 2 events per effective degrees of freedom. Therefore, the estimates from the current models could be unstable and invalid as per the events per variable (EPV) rule (https://doi.org/10.1016/0895-4356(95)00510-2; https://link.springer.com/article/10.1007/s10985-013-9290-4#ref-CR15; https://doi.org/10.1093/aje/kwk052).

   In the previous version, it was recommended to use an alternative method (e.g., propensity score full-matching, propensity score weighting) as a sensitivity analysis for the main findings. Since there are very few events and only 2 events per effective degrees of freedom (i.e., 2 EPV), why should we rely only on the logistic regression results? The odds ratio from the logistic regression also often suffers from many major problems (e.g., non-collapsible bias; https://doi.org/10.1177%2F0962280213505804). The authors should provide a strong rationale why they are insisted (i) using the BDHS 2014 dataset but not the BDHS 2017-18 dataset, (ii) relying only on results from a single model instead of sensitivity analysis of the main findings using an alternative model, and (iii) why EPV is not a major concern, or if EPV is a concern why not they consider multiple cycles to improve statistical power and stability of the model.

2. According to Annex 1, PNC and ENC are mediators in the relationship between place of delivery (exposure) and early neonatal mortality (outcome). Adjusting for a mediator leads to decompose the total effect into direct and indirect effects (https://doi.org/10.1093/ije/dyt127). Therefore, the authors should not adjust for PNC and ENC in the same model when exploring the total effect of delivery place on early neonatal mortality. To explore the effect of PNC and ENC on early neonatal mortality, they should run a separate model.

There are some minor issues as well:

1. The title needs to be changed. Considering the study design of BDHS, one can only explore the association of institutional delivery and newborn care with early-neonatal mortality. We need longitudinal studies to conclude whether institutional delivery and newborn care actually prevent early neonatal deaths. One example revised title is “Exploring the association of the

place of delivery and newborn care with early-neonatal mortality in Bangladesh". Otherwise, please justify why the term "preventing early-neonatal mortality" is appropriate in the title.

2. The study objective in the Introduction is written as "This study aims to explore the association of the place of delivery and newborn care with ENNM in Bangladesh." However, the aim is different in the Abstract. Please restate and clearly specify the study objective in the Abstract.
3. Pleased drop the sentence from the abstract "Singleton births had 95% lower odds of dying in the first seven days of life compared to twin birth (aOR: 0.05; 95% CI: 0.01-0.16) in the same period." This result is not aligned with the study's objective. Instead, it is the Table 2 fallacy.
4. The conclusion in the Abstract is wrong. Since delivery at the health facility is not associated with early neonatal deaths, how the study findings highlight the importance of newborn and postnatal care in preventing early neonatal deaths? Please clarify.
5. The study's strength "The sample size is also relatively large …" is not true. Considering the extremely rare event, the study's sample size ~4000 is tiny (http://www.vanbelle.org/chapters/webchapter2.pdf; https://doi.org/10.1016/0047-2352(86)90111-X). The authors should restate the argument or provide the rationale for using this as a strength.
