# Supplementary material for: Exploring association between place of delivery and newborn care with early-neonatal mortality in Bangladesh
Source: PLoS One. 2022 Jan 27;17(1):e0262408. doi: 10.1371/journal.pone.0262408 (PMC8794140; doi:10.1371/journal.pone.0262408)
Supplement: Attachment — Submitted filename: PONE-D-20-30510 manuscript 27 October 2020.docx [file pone.0262408.s002.docx]

**The role of institutional delivery and newborn care in preventing early-neonatal mortality in Bangladesh**

**Rashida-E Ijdi^1,2*^, Katherine Tumlinson^1,2^, Siân L. Curtis^1,2^**

^1^ Department of Maternal and Child Health, University of North Carolina, Gillings School of Global Public Health, Chapel Hill, NC, USA

^2^ Carolina Population Center, University of North Carolina at Chapel Hill, Chapel Hill, NC, USA.

***Corresponding author:**

E-mail: [ijdi@live.unc.edu](mailto:ijdi@live.unc.edu) and [rashida.ijdi@gmail.com](mailto:rashida.ijdi@gmail.com)

# **ABSTRACT**

**Objective:** Bangladesh achieved the fourth Millennium Development Goal well ahead of schedule, with a significant reduction in under-5 mortality between 1990 and 2015. However, the reduction in neonatal mortality has been stagnant in recent years. The purpose of this study is to identify the factors affecting early neonatal mortality (ENNM), which represents more than 80% of all neonatal mortality in Bangladesh, with a specific focus on place of delivery and newborn care.

**Methods:** In this study, 2014 Bangladesh Demographic and Health Survey data were used for assessing early-neonatal survival in children born in the three years preceding the survey. The role of place of delivery and newborn care in ENNM was examined using multivariate logistic regression models adjusted for clustering and relevant socio-economic, pregnancy, and newborn characteristics.

**Results:** Between 2012 and 2014, there were 4,595 deliveries in 17,863 sampled households, 39% of which were delivered at health facilities. The estimated early neonatal mortality rate during this period was 14 deaths per 1,000 live births. We found that newborns who had received a higher level of essential newborn care components were 57% less likely to die during the first seven days of their lives compared to their counterparts (aOR: 0.43; 95% CI: 0.23-0.80). In addition, newborns who had received any postnatal care were 65% less likely to die in the early neonatal period compared to their counterparts (aOR: 0.35; 95% CI: 0.17-0.70). Facility delivery was not significantly associated with the risk of early newborn death in any of the models.

**Conclusion:** The study findings highlight the importance of newborn and postnatal care in preventing early neonatal deaths. Accelerated improvements are needed to improve the quality of care during and immediately after delivery in health facilities, particularly in facilities serving low-income populations.

# **INTRODUCTION**

Early neonatal mortality (ENNM), defined as the death of a newborn between zero and six days after birth, represents 73% of all neonatal deaths worldwide [1]. As such, the early neonatal period is the most vulnerable time for a child’s survival. ENNM reflects the quality of care received by the mother during the antenatal period and at childbirth [2,3]. The availability, accessibility and quality of skilled birth attendants and emergency obstetric care are considered important for reducing the burden of early-neonatal deaths [3,4].

Globally, the neonatal mortality rate fell by 49%, from 37 deaths in 1990 to 18 deaths per 1,000 live births in 2017, but that decline was slower than the decline in mortality among children aged 1-59 months [5]. In 2018, 2.5 million children died in the first month of life - approximately 7,000 neonatal deaths every day-with one third dying on the first day of life^6^. There also exist marked disparities in neonatal mortality across regions and countries. Regionally, neonatal mortality was highest in sub-Saharan Africa and South Asia, with each estimated at 28 and 26 deaths per 1,000 live births in 2018, respectively [6]. Almost 99% of neonatal deaths occurred in low and middle-income countries [7]. The regional variation of ENNM is shown in **Fig 1** [8].

**Fig 1:** Number of early-neonatal deaths for the five years period preceding the most recent DHS

In Bangladesh, there has been a steady downward trend in childhood mortality with a 46% decline in neonatal mortality and a 65% decline in under-five mortality [9] over the last two decades; as a result, Bangladesh achieved the fourth Millennium Development Goal target ahead of schedule [10]. As the overall rates of child mortality have decreased, child mortality has become increasingly concentrated in the earliest months of life, especially in the early-neonatal period. A comparison of early-neonatal, infant, and under-5 mortality rates in Bangladesh over the last 20 years reveals that ENNM declined at a slower pace than infant and child mortality, with the result that early-neonatal deaths have changed from 30% of all infant deaths in 1993-1994 to 58% in 2010-2014 [9]. To achieve the United Nations’ Sustainable Development Goal (SDG) target 3.2 (reduction of neonatal deaths to at least as low as 12 per 1,000 live births), Bangladesh has to further reduce under-five mortality by 46%, and at the same time also reduce neonatal deaths by 57% [11]. An important next step in meeting the SDG goal for low- and middle-income countries is to provide universal access to skilled care at birth as the key intervention to reduce neonatal mortality [12,13]. To increase access to skilled birth attendants, some countries, including Bangladesh, are attempting to move all deliveries out of home to health institutions (hereafter referred to as health facilities). In Bangladesh, 62% of all births occur at home and most of those are attended by non-skilled birth attendants (for example, traditional birth attendant, unqualified doctors, relatives, or friends) [9].

The main causes of ENNM differ from the causes of deaths of older children. In low- and middle-income countries, like Bangladesh, neonates predominantly die because of preterm birth and intrapartum-related complications and infections, such as sepsis, meningitis, and pneumonia [2,14]. Birth is the time of the highest risk- when more than 40% of maternal deaths (total about 290,000) and stillbirths and early-neonatal deaths (5.5 million) occur every year [13]. In Bangladesh, asphyxia was the most common cause of death (63.8%) for babies who died in the first 24 hours. Overall, infection is the predominant cause of early neonatal and neonatal deaths, comprising 49% and 58% of deaths respectively [14]. Delivery in a health facility with a skilled provider should reduce ENNM, as has been shown in some contexts [15,16]. Facilitating skilled attendance at delivery is thus likely to be a major pathway to improve early neonatal survival [15], albeit not the only one, as access to care for complications occurring after birth, for example neonatal sepsis, is also important. Evidence suggests that, in Bangladesh, as the coverage of facility delivery continues to increase, ensuring a high level of essential newborn care (ENC) use in health facilities will be the key to maintaining the momentum to decrease neonatal mortality [17]. Recent WHO guidelines for newborn health also emphasize the importance of ENC [18].

However, in contexts where most deliveries occur at home, those seeking care at facilities may well be complicated cases, with a higher risk of early neonatal death. In a study using demographic data collected between 1987 and 2005 in Matlab, Bangladesh, maternal and ENNM rates were much higher among women delivering in a health facility, especially in a higher-level facility, than among those delivering at home [19]. Over the course of the period of observation for this study, the percentage of women seeking skilled attendance rose from 5% to more than 50% and, in turn, the relationship between skilled care and maternal death weakened. When uptake of skilled birth attendance or comprehensive obstetric care is low, women will only seek skilled care when they are ill, and they may do so too late for a midwife or doctor to be able to save the lives of the mothers or neonates [19]. Notably, this difference decreased as the percentage seeking skilled delivery care increased over time [19]– which is consistent with the hypothesis that as coverage of pregnancy care increases, more women who are less at risk will seek care; thus, average mortality in those seeking care will decrease. Unfortunately, it is difficult to obtain valid and comparable data on complications for home and facility deliveries in order to ascertain the true relationship between place of delivery and ENNM in Bangladesh. Even in high-income settings, it is impossible to capture all risk factors for complications that direct women towards choosing a facility delivery [20].

It is important to gain a better understanding of whether an increase in facility deliveries will be associated with a concomitant decrease in ENNM. Studies suggest that better monitoring and management of labor, delivery, and the immediate postpartum period are critical to reducing rates of maternal and perinatal mortality (i.e. a stillbirth or early neonatal death) [21,22,23]. Ensuring that labor and the first 24 hours postpartum are managed by a skilled care provider at a healthcare facility is one of the keys to achieving this aim [24]. The purpose of this study is to identify the determinants of ENNM, and its association with facility delivery in Bangladesh. Despite high ENNM in Bangladesh, few studies have been carried out to date focusing on these issues and most of them were small-scale, and clinically oriented [17,19,26,27]. To our knowledge, this is the largest cross-sectional study to investigate the association between place of delivery or receipt of newborn care and ENNM in Bangladesh. This information will assist planners and other collaborators in the health sector to formulate appropriate strategies and interventions to provide good-quality health services and a series of well-timed interventions to further improve newborn and maternal health.

# **METHODOLOGY**

## **Conceptual framework**

In this study, the conceptual framework for ENNM is adapted from Mosley and Chen’s framework for the study of child survival in low- and middle-income countries [28]. This framework seeks to identify factors that directly and indirectly impact the outcome of interest. Our primary outcome is ENNM, which comprises deaths within the first seven days (0-6 days) of life. Potential risk factors for ENNM are depicted in the conceptual framework shown in **Fig 2**.

**Fig 2:** Conceptual framework of early neonatal mortality

The modified conceptual framework groups the independent variables/proximate determinants into three categories: maternal, pregnancy, and early-neonatal factors. While there are a number of distal determinants that could be included in our analysis, for the purpose of this study we chose to focus on two socio-demographic factors: household wealth quintile and place of residence (urban or rural) and one community factor: time to reach to the nearest health facility which is a proxy for distance to the nearest health facility.

We hypothesize that mothers who delivered their children in a facility are less likely to lose their newborns in the first week of life. Based on the literature review, we recognize that the relationship between facility delivery, as the primary exposure, and ENNM, as the outcome, may be confounded by mother’s socio-demographic factors such as mother’s age, education, place of residence, wealth quintile, birth order and time to reach to the nearest health facility. We also identified other pregnancy and delivery care factors such as ANC by medically trained providers, components of ANC, size of child at birth, and sex of the child, which may also act as confounders of this relationship. Other potential secondary exposures are receipt of postnatal care of the child and essential newborn care (ENC) components.

## **Data Source**

The study uses data from the 2014 Bangladesh Demographic and Health Survey (BDHS)- a nationally representative cross-sectional survey using a two-stage stratified random sampling of households. The detailed methodology can be found in the 2014 BDHS report [9]. The survey collected information from 17,863 ever-married women ages 15-49 years who were asked to provide a complete history of all their live births. The BDHS collected information on pregnancy and delivery care for the most recent live births that occurred in the three years preceding the survey, which is our unit of analysis. Cases with missing information on any of the variables used in the models were excluded (309 cases were dropped due to missing values). Thus, in this study, 4,595 live births were included for final analysis, where 64 children died during the early neonatal period.

## **Data analysis**

Outcome variable: Our outcome variable is early-neonatal mortality (ENNM), defined as the death of a live-born child within the first seven days of life (0-6 days) who was born within three years preceding (2012-2014) the BDHS 2014 survey.

Exposure variables: Our primary exposure variable was place of delivery, categorized as home or facility delivery. Facility delivery was defined as births occurring in any health facility including hospitals, health centers, or clinics. Home delivery was defined as giving birth to a child in a residence.

Essential newborn care (ENC) and any infant postnatal care (PNC) were the secondary newborn care exposure variables in this study. (DHS defines an infant as having received any PNC if they receive PNC within 41 days after delivery. However, a majority of infants (88%) who receive PNC receive it within 48 hours of delivery [9]). The ENC variable is a composite indicator derived from the components of ENC measured by the BDHS: use of sterilized instruments to cut the umbilical cord, applying nothing to the cord, immediate drying (within 5 minutes), keeping the baby warm, delaying bathing to 72 hours after birth, and initiating breastfeeding within 1 hour of delivery. Each component was weighted equally and a score was developed for the number of ENC components received by the infant (0-5). The score was then recoded into a binary ENC variable for low (0-2 components) or high (3-5 components) ENC.

Covariates: Covariates included maternal sociodemographic characteristics (age, birth order, education, residence, and wealth), pregnancy care (antenatal care (ANC), attendance by a medically trained provider, and receipt of ANC components), neonatal factors (sex and size of child at birth), and time to reach the nearest health facility. Before conducting logistic regression, covariates that were continuous (e.g. maternal age) or discrete (e.g. birth order) were converted into categorical variables. ANC components measured by BDHS included: blood-pressure taken, urine tested, blood sample taken, weight taken, ultrasonogram conducted, and discussion of dangers signs at least once during any of the ANC visits. Each component was weighted equally, and a score was constructed for the number of ANC components received during the pregnancy (0-6). The score was recoded into a composite ANC variable with categories none, 1-3, 4-5 or all 6 ANC components. Further information on the study variables can be found in the 2014 BDHS report [9].

## **Statistical analysis**

Descriptive analysis was performed to examine the distribution of the variables, and the Pearson’s Chi-square test was used to perform bivariate analysis to test the association between each of the independent variables in the conceptual framework with the main exposure variable, place of delivery (home vs. facility).

Multivariate logistic regression models were used to calculate odds ratios (ORs) and 95% confidence intervals (CIs) to assess the odds of early-neonatal deaths by mother’s pregnancy care and socio-demographic status and exposure to various levels and quality of newborn care.

In the analysis, standard sampling weights were applied to ensure the generalizability of survey results to the national level. Initially, crude ORs were calculated to examine the bivariate relationship between each of the covariates and the main outcome of interest (ENNM). Adjusted ORs (aOR) were then calculated in two final models by adding sociodemographic, pregnancy care factors, and neonatal factors. Variance inflation factors (VIF) were assessed to examine collinearity between variables before entering them into the multivariable models. All analysis was performed using statistical software package STATA version 16.0 [29].

# **RESULTS**

**Table 1** describes the background characteristics of live births in the three years preceding the survey by ENNM status (i.e. death or survival). Male births comprise 53% of all births and 55% of early neonatal deaths in our sample. The majority of births (64%) and deaths (58%) are to women age 20-34. Births to women in the poorest wealth quintile comprise 22% of births but 32% of deaths, while 20% of births and 15% of deaths are among women in the richest wealth quintile. Around three quarters of births and deaths occur in rural areas.

**Table 1:** Background characteristics of the study population, Bangladesh DHS 2014

| **Background characteristics** | **Number of early neonatal deaths^1^** | **Number of children survived from early neonatal period** | **Number of births** |
| --- | --- | --- | --- |
|  | **n (%)** | **n (%)** | **n (%)** |
| **Sex of the child** | | | |
| Male | 35 (54.8) | 2,391 (52.8) | 2,426 (52.8) |
| Female | 29 (45.2) | 2,140 (47.2) | 2,169 (47.2) |
| **Mother’s age at birth** | | | |
| <20 | 24 (37.1) | 1,442 (31.8) | 1,466 (31.9) |
| 20-34 | 37 (57.9) | 2,911 (64.3) | 2,948 (64.2) |
| 35+ | 3 (5.0) | 178 (3.9) | 181 (3.9) |
| **Mother’s educational attainment** | | | |
| No formal education | 6 (9.0) | 644 (14.2) | 650 (14.2) |
| Primary incomplete | 11 (17.4) | 730 (16.1) | 741 (16.1) |
| Primary completed | 10 (15.9) | 532 (11.7) | 542 (11.8) |
| Secondary incomplete | 32 (49.2) | 1,847 (40.7) | 1,878 (40.9) |
| Secondary completed or higher | 5 (8.5) | 778 (17.2) | 784 (17.1) |
| **Wealth quintile** | | | |
| Poorest | 21 (32.3) | 973 (21.5) | 994 (21.6) |
| Poorer | 12 (18.4) | 856 (18.9) | 868 (18.9) |
| Middle | 9 (14.2) | 870 (19.2) | 879 (19.1) |
| Richer | 13 (19.7) | 937 (20.7) | 949 (20.6) |
| Richest | 10 (15.4) | 895 (19.7) | 905 (19.7) |
| **Place of residence** | | | |
| Rural | 49 (76.3) | 3,346 (73.8) | 3,695 (73.9) |
| Urban | 15 (23.7) | 1,185 (26.2) | 1,200 (26.1) |
| **Time to reach to the nearest health facility** | | | |
| 0-15 minutes | 36 (56.0) | 2,375 (52.4) | 2,411 (52.5) |
| 16-30 minutes | 24 (37.1) | 1,712 (37.8) | 1,736 (37.8) |
| More than 30 minutes | 4 (6.9) | 443 (9.8) | 448 (9.7) |
| **Total** | **64** | **4,531** | **4,595** |

^1^ Early neonatal deaths were deaths at age 0-6 days among live-born children.

**Table 2** presents early neonatal outcomes by place of delivery. Overall, 1,769 live births (39%) occurred in a health facility, and 2,826 live births (61%) occurred in a home setting. A total of 64 early neonatal deaths occurred during the study timeframe, for an overall ENNM rate of 13.9 deaths per 1,000 live births. Among all ENN deaths, almost half (44%) of the early neonatal deaths occurred within 24 hours after delivery. The ENNM rate was slightly higher for home deliveries (14.2) than for deliveries at health facilities (13.6).

**Table 2:** Early neonatal outcome (in the three years preceding the survey) by place of delivery, Bangladesh DHS 2014

| **Early Neonatal Mortality (ENNM)** | **Health Facility** | **Home** | **Number of births** |
| --- | --- | --- | --- |
|  | n (%) | n (%) | n |
| Total number of deliveries (%) | 1,769 (38.5) | 2,826 (61.5) | 4,595 |
| **Early neonatal deaths** | **24 (37.5)** | **40 (62.5)** | **64** |
| Early neonatal deaths in 24 hours after delivery | 6 (19.8) | 22 (80.2) | 28 |
| Early neonatal deaths on days ‘1-6’ after birth | 18 (51.3) | 18 (48.7) | 36 |
| **Early Neonatal Mortality rate (per 1000 live births)** | **13.6** | **14.2** | **13.9** |

The proportion of deliveries occurring in health facilities varies significantly according to several variables included in our analysis, as presented in **Table 3**. Facility delivery decreases with increasing birth order and increases as mother’s level of educational attainment increases. Women who lived in rural areas were more likely to deliver at home (69%) than women in urban areas (42%). Facility delivery also increases with increasing wealth. Women who had not received antenatal care from medically trained providers mostly delivered at home (83%), and the percentage of women who delivered in a health facility increases as the number of ANC components received increases. Neonates who did not receive any PNC were mostly delivered at home (81%). Among neonates who received low ENC, 31% were delivered in a health facility compared to 42% of neonates who received high ENC. Almost 46% of women delivered at a health facility when the time to reach to the nearest facility was 0-15 minutes compared to 20% of women who lived more than 30 minutes from a health facility.

**Table 3:** Maternal, pregnancy, and newborn characteristics by place of delivery, Bangladesh DHS 2014

|  | **Home** | **Health Facility** | **Total** | ***p-*value^1^** |
| --- | --- | --- | --- | --- |
|  | (n=2,826) | (n=1,769) | (n=4,595) |  |
| **Mother’s socio-demographic characteristics** | | | | |
| **Maternal age at birth (years)** | | | | |
| <20 | 918 (62.6) | 548 (37.4) | 1,466 | 0.423 |
| 20-34 | 1,788 (60.6) | 1,160 (39.4) | 2,948 |  |
| 35+ | 121 (66.5) | 61 (33.5) | 182 |  |
| **Birth order** | | | | |
| 1 | 957 (51.9) | 888 (48.1) | 1,845 | 0.000*** |
| 2-4 | 1,615 (65.8) | 838 (34.2) | 2,454 |  |
| 5+ | 254 (85.8) | 42 (14.2) | 297 |  |
| **Maternal educational attainment** | | | | |
| No formal education | 546 (84.1) | 104 (15.9) | 650 | 0.000*** |
| Primary incomplete | 566 (76.3) | 175 (23.7) | 741 |  |
| Primary completed | 378 (69.8) | 163 (30.2) | 542 |  |
| Secondary incomplete | 1,094 (58.3) | 784 (41.7) | 1,878 |  |
| Secondary completed or higher | 241 (30.8) | 543 (69.2) | 784 |  |
| **Maternal place of residence** | | | | |
| Rural | 2,325 (68.5) | 1,070 (31.5) | 3,395 | 0.000*** |
| Urban | 501 (41.8) | 699 (58.2) | 1,200 |  |
| **Wealth Quintile** | | | | |
| Poorest | 842 (84.8) | 151 (15.2) | 994 | 0.000*** |
| Poorer | 657 (75.6) | 212 (24.4) | 869 |  |
| Middle | 573 (65.3) | 305 (34.7) | 879 |  |
| Richer | 497 (52.4) | 452 (47.6) | 949 |  |
| Richest | 257 (28.4) | 648 (71.6) | 905 |  |
| **Mother’s pregnancy care characteristics** | | | | |
| **ANC by Medically Trained Providers^2^ (MTPs)** | | | | |
| ANC by non-MTP/no ANC | 1,325 (82.6) | 279 (17.4) | 1,604 | 0.000*** |
| ANC by MTP | 1,502 (50.2) | 1,490 (49.8) | 2,992 |  |
| **ANC components** | | | | |
| No components | 889 (88.2) | 119 (11.8) | 1,008 | 0.000*** |
| 1-3 components | 879 (73.3) | 320 (26.7) | 1,199 |  |
| 4-5 components | 758 (55.4) | 611 (44.6) | 1,369 |  |
| All 6 components | 301 (29.5) | 719 (70.5) | 1,020 |  |
| **Neonatal characteristics** | | | | |
| **Sex of child** | | | | |
| Male | 1,464 (60.3) | 962 (39.7) | 2,426 | 0.126 |
| Female | 1,363 (62.8) | 806 (37.2) | 2,169 |  |
| **Size of child at birth** | | | | |
| Small/very small | 592 (65.1) | 317 (34.9) | 909 | 0.086* |
| Average or larger | 2,235 (60.6) | 1,452 (39.4) | 3,686 |  |
| **Components of Essential Newborn Care (ENC)** | | | | |
| Low ENC | 966 (69.3) | 428 (30.7) | 1,393 | 0.000*** |
| High ENC | 1,860 (58.1) | 1,341 (41.9) | 3,202 |  |
| **Child Postnatal Care (PNC)** | | | | |
| Received no PNC | 1,367 (81.1) | 318 (18.9) | 1,685 | 0.000*** |
| Received any PNC | 1,459 (50.1) | 1,451 (49.9) | 2,910 |  |
| **Community level characteristics** | | | | |
| **Time to reach to the nearest health facility** | | | | |
| 0 - 15 minutes | 1,293 (53.6) | 1,118 (46.4) | 2,411 | 0.000*** |
| 15–30 minutes | 1,174 (67.6) | 562 (32.4) | 1,736 |  |
| More than 30 minutes | 359 (80.2) | 89 (19.8) | 448 |  |

*p<0.10 **p<0.05 ***p<0.001

^1^ Chi-square test

^2^ Medically trained provider (MTP) includes: qualified doctor, nurse/midwife/paramedic, family welfare visitor (FWV), community skilled birth attendant (CSBA), and sub-assistant community medical officer (SACMO).

Results of the logistic regression models are presented in **Table 4**, where model 1 is the crude or unadjusted model, and models 2 and 3 are adjusted for pregnancy and newborn characteristics, respectively. From model 1 we found that child PNC and components of ENC were statistically significant predictors of ENNM without adjusting for other covariates.

**Table 4:** Multivariate logistic regression analysis to estimate the association of early neonatal mortality with maternal sociodemographic, pregnancy care and newborn care characteristics, Bangladesh DHS 2014

|  | **Unadjusted Model 1**  (n=4,595) | **Adjusted Model 2**  (n=4,595) | **Adjusted Model 3**  (n=4,595) |
| --- | --- | --- | --- |
|  | OR (95% CI) | OR (95% CI) | OR (95% CI) |
| **Socio-demographic characteristics** | | | |
| **Mother’s age at birth** | | | |
| <20 | 1.00 | 1.00 | 1.00 |
| 20-34 | 0.77 (0.40 - 1.46) | 1.38 (0.65 - 2.93) | 1.43 (0.66 - 3.09) |
| 35+ | 1.09 (0.30 - 3.93) | 3.25 (0.49 – 21.52) | 3.10 (0.48 – 19.73) |
| **Mother’s educational attainment** | | | |
| No formal education | 0.52 (0.19 - 1.38) | 0.42 (0.12 - 1.45) | 0.41 (0.11 - 1.45) |
| Primary incomplete | 0.89 (0.37 - 2.12) | 0.783 (0.27 - 1.97) | 0.73 (0.26 - 2.01) |
| Primary completed | 1.12 (0.44 - 2.82) | 0.95 (0.37 - 2.45) | 0.99 (0.38 - 2.52) |
| Secondary incomplete | 1.00 | 1.00 | 1.00 |
| Secondary completed or higher | 0.41* (0.14 - 0.18) | 0.35* (0.11 - 0.14) | 0.36* (0.11 - 1.15) |
| **Wealth quintile** | |  |  |
| Poorest | 1.00 | 1.00 | 1.00 |
| Poorer | 0.64 (0.26 - 1.57) | 0.51 (0.20 - 1.26) | 0.50 (0.20 - 1.25) |
| Middle | 0. 49 (0.19 - 1.24) | 0.36** (0.15 - 0.86) | 0.36** (0.14 - 0.91) |
| Richer | 0.63 (0.27 - 1.46) | 0.44* (0.17 - 1.14) | 0.48 (0.18 - 1.28) |
| Richest | 0.51 (0.21 - 1.25) | 0.33** (0.11 - 0.92) | 0.37* (0.13 - 1.05) |
| **Place of residence** | |  |  |
| Rural | 1.00 | 1.00 | 1.00 |
| Urban | 0.87 (0.46 - 1.65) | 0.94 (0.48 - 1.82) | 0.96 (0.49 - 1.87) |
| **Birth order** | |  |  |
| 1 | 1.00 | 1.00 | 1.00 |
| 2-4 | 0.65 (0.35 - 1.19) | 0.48** (0.24 - 0.96) | 0.49* (0.23 - 1.01) |
| 5+ | 0.62 (0.17 - 2.25) | 0.36 (0.05 – 2.61) | 0.38 (0.05 – 2.61) |
| **Pregnancy care characteristics** | | | |
| **ANC by Medically trained Provider (MTP)** | | |  |
| ANC by non-MTP/no ANC | 1.00 | 1.00 | 1.00 |
| ANC by MTP | 1.04 (0.56 - 1.93) | 1.51 (0.49 - 4.93) | 1.69 (0.54 - 5.30) |
| **ANC Components** | |  |  |
| No components | 1.00 | 1.00 | 1.00 |
| 1-3 components | 0.78 (0.35 - 1.73) | 0.58 (0.20 - 1.68) | 0.64 (0.20 - 2.00) |
| 4-5 components | 0.67 (0.28 - 1.55) | 0.54 (0.12 - 2.44) | 0.63 (0.13 - 3.02) |
| All 6 components | 1.11 (0.48 - 2.56) | 1.01 (0.23 - 4.45) | 1.28 (0.28 - 5.89) |
| **Place of delivery** |  |  |  |
| Home | 1.00 | 1.00 | 1.00 |
| Health Facility | 0.95 (0.53 – 1.70) | 0.93 (0.49 – 1.76) | 1.23 (0.62 – 2.45) |
| **Newborn care characteristics** | | | |
| **Sex of the child** | |  |  |
| Male | 1.00 | - | 1.00 |
| Female | 0.92 (0.50 - 1.67) | - | 0.87 (0.47 – 1.62) |
| **Size of child at birth^1^** | |  |  |
| Very small or small | 1.00 | - | 1.00 |
| Average or larger | 0.78 (0.37 - 1.64) | - | 0.87 (0.47 - 1.62) |
| **Components of Essential Newborn Care (ENC)** | | | |
| Low ENC | 1.00 | - | 1.00 |
| High ENC | 0.41** (0.22 - 0.75) | - | 0.43** (0.23 - 0.80) |
| **Child Postnatal Care (PNC)** | |  |  |
| Received no PNC | 1.00 |  | 1.00 |
| Received PNC | 0.41** (0.22 - 0.77) | - | 0.35** (0.17 – 0.70) |
| **Community characteristics** | | | |
| **Time to reach to the nearest health facility** | | | |
| 0 - 15 minutes | 1.00 | 1.00 | 1.00 |
| 16 - 30 minutes | 0.92 (0.49 - 1.71) | 0.78 (0.42 - 1.45) | 0.79 (0.42 - 1.50) |
| More than 30 minutes | 0.65 (0.20 - 2.11) | 0.53 (0.16 - 1.70) | 0.46 (0.13 - 1.60) |

*p<0.10 **p<0.05 ***p<0.001

After adjusting for maternal socio-demographic, and pregnancy characteristics, wealth quintile and birth order were significantly associated with ENNM (model 2). The odds of ENNM were 64% lower for a child born in middle wealth quintile (aOR: 0.36; 95% CI: 0.15-0.86), and 67% lower if the child was born in the richest wealth quintile (aOR: 0.33; 95% CI: 0.11-0.92), compared to a child born in the poorest wealth quintile. Our result shows that neonates of order 2-4 had 52% lower odds of ENNM than the first child (aOR: 0.48; 95% CI: 0.24-0.96). Notably, place of delivery was not significantly associated with ENNM. Mother’s age at birth, education, place of residence, ANC by MTP, ANC components and time to reach the nearest health facility also did not have any statistically significant association with the likelihood of ENNM.

After adjusting for newborn characteristics in model 3, we found that the newborn care variables are associated with significantly lower ENNM. Newborns who received high ENC (i.e. at least 3 ENC components) had 57% lower odds of early neonatal death (aOR: 0.43; 95% CI: 0.23-0.80) than those who received low ENC. The odds of dying in the early neonatal period are 65% lower when the newborn received PNC after birth (aOR: 0.35; 95% CI: 0.17-0.70) compared to their counterparts who did not receive PNC. In this adjusted model, neonates delivered at a health facility had 23% higher odds of ENNM (aOR: 1.23; 95% CI: 0.62-2.45), but this result is not statistically significant. Other findings are similar to model 2 except that the adjusted odds ratio for the wealthiest quintile and birth order 2-4 are now no longer statistically significant at conventional 5% level (*p* = 0.062 and *p =* 0.054, respectively*)*.

# **DISCUSSION**

Using data on 4,595 births in Bangladesh we found 64 deaths within the first week of life yielding an early-neonatal mortality rate of 13.9 per 1,000 live births. The early neonatal mortality rate was 13.6 per 1,000 live births among infants delivered at a health facility and 14.2 per 1,000 live births among those delivered at home. The first 24 hours after delivery are crucial to both newborns and mothers – this study finds that 44% of neonatal deaths occurred during this period. Our analysis identified several factors that are associated with ENNM; however, delivering in a health facility was not significantly associated with the risk of early neonatal death in any of the models.

Selection effects whereby women who experience high-risk pregnancies or complications during delivery may be more likely to deliver in a health facility, likely contribute to early neonatal mortality among facility deliveries, which could attenuate potential protective effects of facility delivery. We are not able to control for differences in such unobserved confounding obstetric risk factors in our analysis. However, recently released data from the 2017-18 BDHS shows that the percentage of births delivered in a health facility increased from 37% in 2014 to 50% in 2017-18 yet the neonatal mortality rate increased from 28 deaths per 1,000 live births in 2014 to 30 deaths per 1,000 live births in 2017-18 [30]. This indicates that, at the population level, higher levels of facility delivery do not necessarily translate into increased survival of newborns, which is consistent with our finding of no association between facility delivery and newborn survival. The quality of delivery and newborn care is an important factor that could contribute to our null findings and to these population-level trends [22,31]. In our analysis, 30% of newborns received low ENC, and 31% of these newborns were delivered in a health facility. According to the 2014 Bangladesh Health Facility Survey (BHFS), only 17% of facilities that provide delivery services performed all seven signal functions for basic emergency obstetric and neonatal care (BEmONC), and only 6% performed all nine signal functions for comprehensive obstetric and neonatal care (CEmONC) [32]. Overall, the preparedness of facilities to provide high quality vaginal delivery care was low, with only 3% of health facilities demonstrating service-specific readiness in terms of WHO-recommended minimum requirements for quality services [33]. Lack of trained staff, guidelines, life-saving medicines and partograph was more pronounced than the lack of other essential items for providing quality normal delivery services [32]. Facility deliveries will not save newborn lives if health facilities are not able to provide life-saving care in an emergency. This phenomenon was also seen in other South Asian countries [34].

Existing evidence indicates that essential newborn care practices can contribute to reducing early-neonatal morbidity and mortality in Bangladesh [17,35,36]. Our analysis also found that a higher reported level of essential newborn care is significantly associated with increased early newborn survival. A review of recent research found that a significant proportion of neonates born at home in low-and-middle income countries experienced hypothermia, which contributed as a comorbidity to major causes of neonatal deaths such as infection, asphyxia, and preterm birth [19]. Essential newborn care can address these causes of early neonatal mortality in infants. In our study 30% of all newborns received less than three components of ENC and this was higher among infants born at home (21%) compared to infants born in a health facility (9%). Recent study findings have shown that women can not necessarily recall exact timing or sequence of events (e.g. components of ENC; PNC etc.) during or immediately after delivery, leading to recall bias in newborn care indicators, which needs to be kept in mind when interpreting these results [37]. Nevertheless, our study provides evidence that there is scope to improve the quality of ENC among both home and facility births and that such improvement could reduce ENNM in Bangladesh.

Postnatal care provides an opportunity to assess and treat complications in newborns and to counsel mothers on how to care for themselves and their newborns. We found that infants who reportedly received any PNC experienced significantly lower early neonatal mortality than those who did not, consistent with other studies in Bangladesh and neighboring countries [36,38,39]. One caveat in interpreting these results, however, is that those infants who die immediately around the time of delivery will not survive long enough to receive PNC. We reran the models excluding infants who died in the first 24 hours after delivery and found PNC was still highly significant. Most PNC first occurs within the first 48 hours after delivery when it is reported. We found that among all live births, 30% of infants born at home and 7% of infants born in health facilities were reported to have not received any PNC; increasing access to high-quality early PNC remains an important intervention to reduce ENNM.

Our study found that ENNM is higher in poor households. Poor households often not only lack the resources required to access recommended antenatal and postnatal checkups [40] and high-quality delivery care, highlighting the need for continued attention to equity in access to maternal and newborn care in Bangladesh but also to larger environmental, social and structural factors that result in higher risks for infants born to mothers from low-income households.

We did not find any statistically significant effect of birth size on mortality. This is counterintuitive, given that birth size is a proxy measure of birth weight, which is an important factor in ENNM due to its association with intrauterine growth retardation and prematurity [41,42,43]. A possible reason for this finding is the low validity of the size of the child at birth variable, which is based on the mother’s self-report in DHS. Other studies also failed to establish a significant association between size at birth and neonatal mortality using DHS data [43].

## **Strengths and limitations**

This study has several limitations, some of which have already been touched on above. The reliability of mortality estimates calculated from retrospective birth histories depends upon the extent to which birth dates and ages at death are accurately reported and recorded. Omission of either births or deaths is a serious problem since they affect the accuracy of the mortality estimates. Errors in reporting of birth dates may cause a distortion of trends over time, while errors in reporting of age at death can distort the age pattern of mortality. Omission of early neonatal deaths is of particular concern in DHS birth history data [9] and could lead to under-estimation of ENNM in this study [9,44]. Second, as noted above, newborns must survive long enough to receive PNC so some instances of lack of newborn care may be due to early neonatal death rather than the other way round. Third, there is known recall bias in women’s reports of both maternal and newborn care at the time of delivery [45]. Finally, information on pregnancy, and delivery complications were not captured in the BDHS 2014 so we are unable to control for obstetric risk factors that could affect both ENNM and the choice to deliver in a health facility.

Despite these limitations, this study has many strengths. The BDHS data are nationally representative and have a high (98%) response rate; as a result, sample selection bias is unlikely to affect the study findings. The BDHS used a well-tested, standardized questionnaire and employed a high-quality data collection process to reduce interview bias and to identify and address field data collection problems rapidly. The sample size is also relatively large, which is particularly important when studying an event like early neonatal mortality that is relatively rare.

# **CONCLUSION**

This study provides evidence on ENNM risk factors for national policy makers and health experts who aim to reduce ENNM in Bangladesh. Our study findings indicate that increasing facility delivery is not sufficient to reduce ENNM by itself. Increased attention to the quality of care and equitable access to high-quality obstetric care is required if increases in facility delivery are to result in gains in early neonatal survival. Comprehensive ENC and PNC have significant influences on early newborn survival. Standard operating procedures (SOPs) to ensure the quality of care during delivery, to ensure all components of ENC, and to provide high quality early postnatal care are of critical importance to realize the theoretical potential of facility delivery to avert neonatal deaths.

**Abbreviations:** ANC, antenatal care; aOR, adjusted odds ratio; BDHS, Bangladesh demographic and health survey; BEmONC, basic emergency obstetric and neonatal care; BHFS, Bangladesh health facility survey; CEmONC, comprehensive emergency obstetric and neonatal care; CI, confidence interval; ENNM, early neonatal mortality; ENC, essential newborn care; PNC, postnatal care; SDG, sustainable development goals; SOP, standard operating procedure; VIF, variance inflation factors; WHO, World Health Organization.

**Author contributions:** Conceptualization **–** RI SC KT. Formal analysis: RI SC. Original draft preparation: RI. Review & Editing: SC KT. All authors read and approved the final manuscript.

**Acknowledgments:** The authors would like to thank the DHS Program for granting access to BDHS datasets and making publicly available the published DHS reports. The views expressed herein are solely those of the authors and do not necessarily reflect the views of any institution or organization.

**Ethics approval and consent to participate:** BDHS data are public access data and were made available to us by MEASURE DHS upon request. While conducting data collection, an informed consent was obtained from the respondents prior to the interview. Ethical clearance to conduct the BDHS was approved by the Government of Bangladesh. IRB approval was granted (on March 30, 2020) by the University of North Carolina at Chapel Hill, USA before proceeding with this analysis.

**Data availability statement:** The authors confirm that all data underlying the findings are available without restriction. The data sources used in the analysis can be accessed at <https://www.dhsprogram.com/data/available-datasets.cfm>.

**Funding:** The author(s) received no specific funding for this work.

**Competing interests:** The authors have declared that no competing interests exist.

# **REFERENCE**

1. Lehtonen L, Gimeno A, Parra-Llorca A, Vento M. Early neonatal death: A challenge worldwide. Seminars in Fetal & Neonatal Medicine. 2017; 22(2017)153-160. Doi: [https://doi.org/10.1016/j.siny.2017.02.006](https://doi-org.libproxy.lib.unc.edu/10.1016/j.siny.2017.02.006)
2. Ngoc N, Merialdi M, Aleem H, Carroli G, Purwar M, Zavaleta N. Causes of stillbirths and early neonatal deaths: data from 7993 pregnancies in six developing countries. Bulletin of the World Health Organization. September 2006; 84(9): 699-705.
3. World Health Organization (WHO). Neonatal and Perinatal Mortality – Country, Regional and Global Estimates. World Health Organization. 2006, Geneva, Switzerland.
4. Lawn JE, Lee A, Kinney M, Sibley L, Carlo WA, Paul VK, et al. Two million intrapartum-related stillbirths and neonatal deaths: Where, why, and what can be done? International Journal of Gynecology and Obstetrics. 2009; 107 (S5-S19).
5. United Nations Population Division. Level and Trends in Child Mortality Report 2018. Department of Economic and Social Affairs. Available from: <https://www.un.org/en/development/desa/population/publications/mortality/child-mortality-report-2018.asp>.
6. United Nations Children’s Fund (UNICEF). Neonatal mortality. The United Nations Inter-agency Group for Child Estimation (UN IGME). 2019. Available from: <https://data.unicef.org/topic/child-survival/neonatal-mortality/>.
7. Belizan JM, McClure M, Goudar S, Pasha O, Esamai F, Patel A. et al. Neonatal Deathin Low-to Middle-Income Countries: A Global Network Study. American Journal of Perinatology. 2012; 29(8).
8. ICF. The DHS Program STATcompiler. Funded by USAID. http://www.statcompiler.com.
9. National Institute of Population Research and Training (NIPORT), Mitra and Associates, and ICF International 2016. Bangladesh Demographic and Health Survey 2014. NIPORT and ICF International. Dhaka, Bangladesh and Rockville, Maryland. 2016. Available from: <https://dhsprogram.com/pubs/pdf/FR311/FR311.pdf>.
10. Government of the People’s Republic of Bangladesh. Millennium Development Goals: End-period Stocktaking and Final Evaluation Report (2000-2015). General Economic Division (GED), Bangladesh Planning Commission. Published. September 2016.
11. National Institute of Publication Research and Training (NIPORT), and ICF International. Bangladesh Demographic and Health Survey 2014: Policy Briefs. NIPORT and ICF International. Dhaka, Bangladesh and Rockville, Maryland. 2016.
12. WHO, UNICEF. Every Newborn: an action to end preventable deaths: Executive summary. Whorl Health Organization. Geneva, Switzerland. 2014.
13. Lawn JE, Blencowe H, Oza S, You D, Lee AC, Waiswa P. et al. Every Newborn: progress, priorities, and potential beyond survival. Lancet. 2014 Jul 12;384(9938):132.
14. Halim A, Dewez JE, Biswas A, Rahman F, White S, Broek NVD. When, Where, and Why Are Babies Dying? Neonatal Death Surveillance and Review in Bangladesh. PLoS ONE 2016.11(8): e0159388.
15. Malqvist M, Sohel N, Do TT, Eriksson L, Persson LA. Distance decay in delivery care utilization associated with neonatal mortality. A case referent study in northern Vietnam. BMC Public Health. 2010. 10: 762.
16. Titaley CR, Dibley MJ, Roberts CL. Type of delivery attendant, place of delivery and risk of early neonatal mortality: analyses of the 1994–2007 Indonesia Demographic and Health Surveys. Health Policy Plan. August 2012;27(5):405-16. Doi:10.1093/heapol/czr053.
17. Kim ET, Singh K. The State of Essential Newborn Care by Delivery Location in Bangladesh. Maternal Child Health J. 2017; 21(11):2078-2085.
18. WHO recommendations on newborn health: guidelines approved by the WHO Guidelines Review Committee. Geneva: World Health Organization; 2017 (WHO/MCA/17.07). Licence: CC BY-NC-SA 3.0 IGO.
19. Ronsmans C, Chowdhury ME, Koblinsky M, Ahmed A. Care seeking at time of childbirth, and maternal and perinatal mortality in Matlab, Bangladesh. Bull World Health Organ. 2010. 88: 289–296.
20. Birthplace in England Collaborative Group. Perinatal and maternal outcomes by planned place of birth for healthy women with low risk pregnancies: The Birthplace in England national prospective cohort study. BMJ 2011; 343:d7400. Available from: doi:  [https://doi.org/10.Stein C. 1136/bmj.d7400](https://doi.org/10.Stein%20C.%201136/bmj.d7400)
21. Lawn J, Shibuya K, Stein C. No cry at birth: global estimates of intrapartum stillbirths and intrapartum-related neonatal deaths. Bull World Health Organ 83: 409–417.
22. Ronsmans C, Graham WJ. Maternal mortality: who, when, where and why. Lancet Maternal Survival Series Steering Group. Lancet. 2006; 368:1189–200. doi:10.1016/S0140-6736(06)69380-X.
23. Baqui AH, Darmstadt, Williams EK, Kumar V, Kiran TU, Panwar D. et al. Rates, timing and causes of neonatal deaths in rural India: implications for neonatal health programmes. Bulletin of the World Health Organization. September 2006; 84(9).
24. Campbell OMR, Grahan W, Ronsmans C, Borghi J. et al. Maternal Survival 2: Strategies for reducing maternal mortality: getting on with what works. The Lancet; October 2006; 368, 9543; ProQuest pg. 1284.
25. Kunkel M. Marete I, Cheng E, Bucher S, Liechty E, Esamai F. Place of delivery and perinatal mortality in Kenya. Elsevier: Seminars in Perinatology. 2019; 43. 252-259.
26. Fottrell E, Osrin D, Alcock G, Azad K, Bapat U, Beard J. et al. Cause-specific neonatal mortality: analysis of 3772 neonatal deaths in Nepal, Bangladesh, Malawi and India. Global Child Health. 2015; 100:F439-F447. doi:10.1136/archdischild-2014-307636.
27. Abir T, Ogbo FA, Stevens GJ, Page AN, Milton AH, Agho KE. The impact of antenatal care, iron-folic acid supplementation and tetanus toxoid vaccination during pregnancy on child mortality in Bangladesh. PLOS One. 2017.12(11):e0187090.
28. Kibria GM, Burrowes V, Choudhury A, Sharmeen, Ghosh S, Mahmud A. Determinants of early neonatal mortality in Afghanistan: an analysis of the Demographic and Health Survey 2015. Globalization and Health. 2018;14:47.
29. StataCorp. Stata Statistical Software: Release 15. College Station, Tx: StataCorp LLC; 2017.
30. National Institute of Population Research and Training (NIPORT), and ICF. Bangladesh Demographic and Health Survey 2017-18: Key Indicators. Dhaka, Bangladesh, and Rockville, Maryland, USA: NIPORT, and ICF. 2019.
31. Broek NR, Graham WJ. Quality of care for maternal and newborn health: the neglected agenda. BJOG 2009; 116 Suppl1:18-21.
32. National Institute of Population Research and Training (NIPORT), Association for Community and Population Research (ACPR), and ICF International. 2014 Bangladesh Health Facility Survey (BHFS). Dhaka, Bangladesh: NIPORT, ACPR and ICF International. 2016.
33. World Health Organization (WHO) Service availability and readiness assessment (SARA): an annual monitoring system for service delivery, reference manual. 2013. Version 2.1. Available from: <http://www.who.int/healthinfo/systems/SARA_Reference_Manual_Full.pdf>.
34. Fariyal F. Fikree, Ali M. Mir, and Inaam-ul Haq, “She may reach a facility but will still die! An analysis of quality of public sector maternal health services, District Multan, Pakistan,” Journal of the Pakistan Medical Association 56, 4. 2006 : 156-63.
35. Bhutta ZA, Das JK, Bahl R, et al. Can available interventions end preventable deaths in mothers, newborn babies, and stillbirths, and at what cost? Lancet 2014;384:347-70. Doi: 10.1016/S0140-6736(14)60792-3.
36. Akter T, Dawson A, Sibbritt D. What impact do essential newborn care practices have on neonatal mortality in low and lower-middle income countries? Evidence from Bangladesh. Journal of Perinatology. 2016; 36,225-230.

37. Blanc AK, Warren C, McCarthy KJ, Kimani J, Ndwiga C, RamaRao S. Assessing the validity of indicators of the quality of maternal and newborn health care in Kenya . Journal of Global Health. 2016; vol6.1.010405.

38. Bhutta ZA, Memon ZA, Soofi S, Salat MS, Cousens S, et al. Implementing community-based perinatal care: results from a pilot study in rural Pakistan. Bull World Health Organ. 2008; 86(6): 452-459.

39. Kumar V, Mohanty S, Kumar A, Misra RP, Santosham M, et al. Effect of community-based behaviour change management on neonatal mortality in Shivgarh, Uttar Pradesh, India: a cluster randomized controlled trail. The Lancet. 2008; 372(9644): 1151-1162.

40. Gogia S, Sachdev HS. Home visits by community health workers to prevent neonatal deaths in developing countries: a systematic review. Bull World Health Organ. 2010; 88(9): 658B-666B.

41. UNICEF. Committing to Child Survival: A Promise Renewed – Progress Report 2014. United Nations Children’s Fund. September 2014. ISBN: 978-92-806-4770-9.

42. Katz J, Lee AC, Kozuki N, Lawn JE, Cousens S, et al. Mortality risk in preterm and small-for-gestational-age infants in low-income and middle-income countries: a pooled country analysis. The Lancet. 2013; 382(9890): 417-425.

43. Flendy V, Middleton P, Smith G, Duke W, Erwich J, Khong T. et al. Stillbirths: the way forward in high-income countries. The Lancet. 2011;377:173-17.

44. Pullum TW, Becker S. Evidence of Omission and Displacement in DHS Birth Histories. DHS Methodological Reports No. 11. Rockville, Maryland, USA: ICF International. 2014.

45. Hossain B, Mistry SK, Mohsin M, Khan MHR. Trends and determinants of perinatal mortality in Bangladesh. Plos One. 2019.
