# Supplementary material for: Exploring association between place of delivery and newborn care with early-neonatal mortality in Bangladesh
Source: PLoS One. 2022 Jan 27;17(1):e0262408. doi: 10.1371/journal.pone.0262408 (PMC8794140; doi:10.1371/journal.pone.0262408)
Supplement: Attachment — Submitted filename: Response to Reviewers_25Aug2021.docx [file pone.0262408.s007.docx]

PONE-D-20-30510R1

Title of the paper: The role of institutional delivery and newborn care in preventing early-neonatal mortality in Bangladesh

**Reviewer’s #2**

I am repeating my comments that I submitted as an attachment:

The authors have made some significant changes. However, there are still many major issues that need to be addressed:

**1.** In response to aggregating 2/3 cycles of data to improve statistical power, the authors mentioned that the proportion of women delivering in health facilities has been increasing over time, affecting the relationship between delivery care and early neonatal mortality. If this is true, why would we believe findings from BDHS 2014 data would reflect the current scenario of the association? The BDHS 2017-18 dataset is already publicly available, and it should only take a couple of days to access the dataset. Then why would be we believe BDHS 2014 dataset is more likely to reflect the current scenario than BDHS 2017-18?

In Table 4 (model 3), the authors adjusted the model for many covariates. However, the number of events is only 69 in the study (Table 1). For example, there are only 69/29 ≈ 2 events per effective degrees of freedom. Therefore, the estimates from the current models could be unstable and invalid as per the events per variable (EPV) rule (https://doi.org/10.1016/0895-4356(95)00510-2; https://link.springer.com/article/10.1007/s10985-013-9290-4#ref-CR15; https://doi.org/10.1093/aje/kwk052).

In the previous version, it was recommended to use an alternative method (e.g., propensity score full-matching, propensity score weighting) as a sensitivity analysis for the main findings. Since there are very few events and only 2 events per effective degrees of freedom (i.e., 2 EPV), why should we rely only on the logistic regression results? The odds ratio from the logistic regression also often suffers from many major problems (e.g., non-collapsible bias; https://doi.org/10.1177%2F0962280213505804). The authors should provide a strong rationale why they are insisted (i) using the BDHS 2014 dataset but not the BDHS 2017-18 dataset, (ii) relying only on results from a single model instead of sensitivity analysis of the main findings using an alternative model, and (iii) why EPV is not a major concern, or if EPV is a concern why not they consider multiple cycles to improve statistical power and stability of the model.

Answer: Our justification: We submitted our manuscript on 29 September 2020, roughly two and half months before the public release of the latest BDHS (i.e., 2017-18 round) data. We received our first round of review on 14 January 2021, which recommended redoing the analysis using the latest BDHS round. We respectfully disagreed with that comment, mentioning that re-analyzing with the BDHS 2017-18 data fell outside the scope of the manuscript. Given the timing of our initial submission and the amount of effort that has been put to prepare the manuscript, we would like to re-iterate our position regarding this manuscript. We do not believe that the data from 2014 have become outdated, as a Google Scholar search indicates that nearly 20 articles were published in the last one year using these data, including one published in PLOS One in July 2021 (doi: 10.1371.journal.pone.0254777).

We thank the PLOS ONE for accepting our justification to use the 2014 BDHS data for this manuscript.

**2.** According to Annex 1, PNC and ENC are mediators in the relationship between place of delivery (exposure) and early neonatal mortality (outcome). Adjusting for a mediator leads to decompose the total effect into direct and indirect effects (https://doi.org/10.1093/ije/dyt127). Therefore, the authors should not adjust for PNC and ENC in the same model when exploring the total effect of delivery place on early neonatal mortality. To explore the effect of PNC and ENC on early neonatal mortality, they should run a separate model.

Answer: We would like to thank the reviewers for this comment. We have modified the DAG accordingly; please see the updated DAG in Supplementary Information “S1 Figure”.

There are some minor issues as well:

1. The title needs to be changed. Considering the study design of BDHS, one can only explore the association of institutional delivery and newborn care with early-neonatal mortality. We need longitudinal studies to conclude whether institutional delivery and newborn care actually prevent early neonatal deaths. One example revised title is “Exploring the association of the place of delivery and newborn care with early-neonatal mortality in Bangladesh”. Otherwise, please justify why the term “preventing early-neonatal mortality” is appropriate in the title.

Answer: We would like to thank the reviewer for this comment. We have changed the title of the paper as “Exploring association between place of delivery and newborn care with early-neonatal mortality in Bangladesh”.

2. The study objective in the Introduction is written as “This study aims to explore the association of the place of delivery and newborn care with ENNM in Bangladesh.” However, the aim is different in the Abstract. Please restate and clearly specify the study objective in the Abstract.

Answer: We thank the reviewer for this comment. The abstract has been updated accordingly, as “The purpose of this study is to explore the association between place of delivery and newborn care with early neonatal mortality (ENNM)”, in page 1, lines 4-6.

3. Pleased drop the sentence from the abstract “Singleton births had 95% lower odds of dying in the first seven days of life compared to twin birth (aOR: 0.05; 95% CI: 0.01-0.16) in the same period.” This result is not aligned with the study’s objective. Instead, it is the Table 2 fallacy.

Answer: We would like to thank the reviewer for this comment. We have dropped the sentence “Singleton births ….. in the same period” from the abstract.

4. The conclusion in the Abstract is wrong. Since delivery at the health facility is not associated with early neonatal deaths, how the study findings highlight the importance of newborn and postnatal care in preventing early neonatal deaths? Please clarify.

Answer: We have modified the conclusion accordingly, in page 2, lines 2-6, as “Further, findings suggest that increasing the proportion of women who give birth in a healthcare facility is not sufficient to reduce ENNM by itself; to realize the theoretical potential of facility delivery to avert neonatal deaths, we must also ensure quality of care during delivery, guarantee all components of ENC, and provide high-quality early PNC.”

5. The study’s strength “The sample size is also relatively large …” is not true. Considering the extremely rare event, the study’s sample size ~4000 is tiny (http://www.vanbelle.org/chapters/webchapter2.pdf; https://doi.org/10.1016/0047-2352(86)90111-X). The authors should restate the argument or provide the rationale for using this as a strength.

Answer: We would like to thank the reviewer for this comment. We have restated the argument as a limitation at page 22, lines 7-9.

**Reviewer # 3**

I have gone through the revised manuscript and found insufficient responses to some vital points. Hence, still following questions must be responded properly to improve this manuscript.

1. Authors need to give more strong reference against their claim “Based on best practice, our reference is to include only the minimally sufficient adjustment set" as mentioned in the answer of first point. Many studies found direct associations of parental education, employment status, occupation with neo-natal mortality.

Otherwise, they need to carry out the analysis with these variables in the model.

Answer: As we have mentioned, in our first revision we have already included paternal education. As a reminder, we define a variable as a confounder if it is associated with both the exposure and the outcome and not on the causal pathway, as is best practice in the field of epidemiology. Because we do not believe that paternal employment status and occupation are associated with place of delivery, ENC, or PNC, we cannot include these variables in our regression model.

2. The data of BDHS2018 is available and all the variable codes are same as BDHS2014. Authors must need to redo all analyses with this data. Authors mentioned that they will do the same analysis and make another paper which is pointless as the dataset is already available.

Answer: According to the editor’s decision we have decided to keep the analysis of the paper using BDHS 2014 data.

3. Authors did not mention whether there was no any missingness in the covariates considered apart from answering missingness among the discarded 309 cases.

Answer: In the first round of review, regarding missing values we have already mentioned that “The 309 cases were in fact dropped because they were not the most recent live birth, thus ANC, PNC information were not available.”

4. Figure 1 in previous manuscript was suggested to revise by using MapReasy which is important to strengthen this manuscript but authors omitted this. Authors need to generate and include such graph.

Answer: We have dropped the map (Figure 1) according to editor’s comment in the first round of review.

5. Authors still mentioned in abstract and statisticcal analysis section that multivariate logistic..... It should be multivaiable logistic....

Answer: We have carefully checked the manuscript and we do not find any remaining use of the term ‘multivariate logistic.’

6. Some references are missing pages, volume, series etc. E.g., references 18, 36, 41, 43.

Answer: We would like to thank the reviewer for the very careful review. We have checked and confirming that references 18, 36, 41 are properly cited. We have added the volume number and electronic identifier (uses by PLOS ONE instead of page numbers) for reference # 43.

7. Study strengths were unnecessarily been mentioned in the last section. Only limitations are OK.

Answer: We would like to thank the reviewer for this comment. We have decided to keep both strengths and limitations in this manuscript.

8. Page 6, l12: DAG has been created and a minimally sufficient adjustment is set according to ref 27. There are many published well known studies but authors cited ref 27 which is not a published paper and not worthy. Here how had DAG been created?

Answer: We have referred a textbook of epidemiology, which we found appropriate here. Respectfully, Drs. Kenneth Rothman, Sander Greenland, and Tim Lash and the content of their book, Modern Epidemiology, are more than ‘worthy’ to be cited to justify use of a DAG to select a minimally sufficient adjustment set.

9. Data source is insufficiently discussed.

Answer: We have specified the data source in page 6, lines 16-18, as “The study uses data from the 2014 Bangladesh Demographic and Health Survey (BDHS)- a nationally representative cross-sectional survey using a two-stage stratified random sampling of households, which covered all districts and administrative divisions of Bangladesh.”

**Editor’s comment:**

Reviewers have raised valid concerns regarding data analysis and the dataset analyzed. Please consider analyzing the latest DHS data to provide the most updated information.

Answer: According to the editions mail (dated August 19, 2021) mentioned “the use of the 2014 dataset is justified given that the manuscript was submitted before the public release of the latest BDHS data.” Thus, we are keeping the analysis in this paper using the BDHS 2014 data.
